# The Role of Cognitive Emotion Regulation Strategies in Problem Gaming Among Adolescents: A Nationally Representative Survey Study

**DOI:** 10.3389/fpsyt.2019.00273

**Published:** 2019-04-29

**Authors:** Gyöngyi Kökönyei, Natália Kocsel, Orsolya Király, Mark D. Griffiths, Attila Galambos, Anna Magi, Borbála Paksi, Zsolt Demetrovics

**Affiliations:** ^1 ^Institute of Psychology, ELTE Eötvös Loránd University, Budapest, Hungary; ^2^SE-NAP2 Genetic Brain Imaging Migraine Research Group, Hungarian Academy of Sciences, Semmelweis University, Budapest, Hungary; ^3^Department of Pharmacodynamics, Faculty of Pharmacy, Semmelweis University, Budapest, Hungary; ^4^Doctoral School of Psychology, ELTE Eötvös Loránd University, Budapest, Hungary; ^5^Psychology Department, Nottingham Trent University, Nottingham, United Kingdom; ^6^Institute of Education, ELTE Eötvös Loránd University, Budapest, Hungary

**Keywords:** online gaming, rumination, positive reappraisal, emotion regulation, adolescent gaming, gender differences

## Abstract

Explanatory theoretical models have proposed an association between problematic online gaming and abilities or strategies in alleviating distress or negative emotions in times of stress as proximal non-gaming-related personality factors. However, there is little research that has targeted how emotion regulation relates to problematic online gaming—especially during adolescence when gaming behavior is most prevalent. In emotion regulation research, there has been a particular emphasis on rumination because it is strongly associated with overall psychopathology. However, it is unknown whether this putatively maladaptive strategy relates to problematic online gaming and whether it is a gender-dependent association. Consequently, the present study examined how emotion regulation strategies, and particularly rumination, related to problem gaming and tested whether gender moderated this relationship in adolescents. In a national representative adolescent sample, 46.9% of the participants (*N* = 1,646) reported online gaming in the past 12 months and provided information on problematic gaming, and it was these data that were used for further analysis. Their data concerning problematic online gaming and emotion regulation strategies were analyzed, including rumination along with other putatively maladaptive (e.g., catastrophizing) and adaptive (e.g., positive reappraisal) strategies, while controlling for age, gender, and game genre preference. Results of linear regression analyses showed that all the putatively maladaptive emotion regulation strategies (including self-blame, other blame, catastrophizing, and rumination) were positively related to problematic online gaming. Positive reappraisal proved to be a protective factor; it was inversely related to problematic online gaming. In addition, the relationship between rumination and online gaming was moderated by gender (i.e., the relationship was stronger among boys). Based on the results, it is argued that emotion regulation is a useful framework to study problematic online gaming. The present study highlighted that the relative predictive value of rumination for problematic online gaming varied for boys and girls, suggesting that trait rumination might be a gender-specific vulnerability factor for problematic online gaming, but this requires further investigation and replication.

## Introduction

Online gaming is a widespread phenomenon in adolescence and many teenagers are involved in this leisure activity on a regular basis. Recent data from the European School Survey Project on Alcohol and Other Drugs that used representative samples (1) indicated that more than 20% of adolescents (aged 16 years old) engaged in frequent online gaming (defined as at least four times in the past 7 days). Most teenagers engage in gaming simply for fun, enjoyment (2), social reasons [e.g., friendship (3, 4)], mastery, and/or competition-related motives [see Ref. (5)], and often moderated by game genre (game type) (6, 7). Playing videogames has both beneficial effects (8) and costs (9), and for a small minority of individuals, it can become problematic, negatively affecting everyday life.

Although there is a debate on how problematic online gaming—or internet gaming disorder (IGD) as introduced into Section 3 of the fifth edition of the *Diagnostic and Statistical Manual of Mental Disorders* (*DSM-5*)—should be conceptualized (10, 11), there is some consensus. Gaming is perhaps best characterized as a continuum ranging from non-problem gaming (non-regularly, regularly, and even excessively) to problem and addictive gaming at the other end (12, 13). The accurate assessment and prevalence of problem gaming is challenging due to the overpathologizing nature of the screening instruments [see Ref. (14)]. However, it still affects a considerable number of adolescents. According to studies involving representative samples, the prevalence is estimated to be between 1% (15–18) and 9% (19).

Problem gaming is associated with psychiatric symptoms (20, 21), psychosomatic complaints (22), and poorer academic achievements (23). In addition, it appears to be relatively stable over time among fourth- and fifth-grade students (24) and adolescents aged 13 years and above (20, 23). These data strongly suggest that identifying both distal and proximal gaming and non-gaming-related factors (e.g., personality, family, peer, genetic factors, etc). that are associated with problem gaming—concurrently or longitudinally—should be a high priority, along with demonstrating those factors that separate non-problem gamers from problem and/or addicted gamers.

High engagement indexed by preoccupation and tolerance, for instance, might not be a sufficient criterion in distinguishing pathological and recreational gaming (25, 26). Similarly, intense gaming indexed by gaming time has been questioned as being a reliable predictor of problem gaming (25, 27). These findings are not surprising given that gamers have very different explicit (28) and implicit (29) motives to play that may vary as a function of their mood (30), emotions (5), and psychological needs (31, 32). These empirical findings suggest that online gaming may serve regulatory goals and highlight that identification of underlying reasons why a specific individual in a specific context engages in online gaming is warranted (33). For example, Kardefelt-Winther (34) proposed investigating compensatory functions of online space use to elucidate factors that distinguish between healthy and problematic online gaming. Indeed, studies suggest that gaming motivations to escape from real-life problems and difficulties act as mediators between psychiatric symptoms and problem gaming (21). It is worth noting that temporary distraction from real-life problems itself should not be considered as maladaptive. Avoidance is a risk factor for psychopathologies only if it becomes a habitual strategy to handle negative emotions (35, 36).

Explanatory theoretical models other than compensatory internet use, such as the Interaction of Person–Affect–Cognition–Execution (I-PACE) model by Brand et al. (37) and the cognitive–behavioral model by Dong and Potenza (38), also emphasize that alleviating negative emotions or boosting positive ones as potential experienced consequences of gaming (i.e., positive and negative reinforcements) has the potential to contribute to the development and maintenance of problem gaming. Paulus and colleagues (39) in their review on IGD in children and adolescents also list difficulties in coping with negative emotions among personal factors that may contribute to problem gaming. Therefore, it appears reasonable to assume that those who struggle with regulating their emotions or use putatively maladaptive emotion regulation (ER) strategies in times of stress are more at risk for developing problem gaming use. Indeed, among youth aged between 13 and 21 years, ER difficulties—namely, lack of emotional clarity and lack of impulse control in face of negative emotions—have been associated with problem gaming (40). Studies targeting adolescents show that problem gamers use avoidance-related emotion-focused strategies, such as denial, behavioral disengagement, and media use to cope with stress (22, 41).

It is worth noting that ER difficulties or coping strategies that alter emotional responses have been related to not only problem gaming but also studies examining problematic internet use (22, 40), gambling, and substance use particularly (40). These findings support the contention that dysregulated negative emotions are non-specific risk factors across several psychopathologies. Clinical utility and the transdiagnostic nature of ER have been proposed in many studies and reviews (42–44). Furthermore, gambling disorder (as a behavioral addiction) has been theorized within an ER framework (45), using the extended process model of ER (46).

ER refers to any implicit or explicit processes or strategies (47, 48) that act during emotion generation to alter the timing, intensity, duration, and/or valence of emotional experience or expression (49, 50). This definition implies that ER can be operationalized and studied in many ways. For example, many studies have examined how self-reported ER strategies (51, 52) and experimentally induced strategies (53, 54)—often grouped into adaptive or maladaptive ones—account for emotional or behavioral functioning. Self-reported use of putatively maladaptive ER strategies, including rumination, suppression, and avoidance, has been shown to be reliably associated with psychopathological symptoms (42, 51). Findings concerning the protective role of putatively adaptive strategies including positive reappraisal or acceptance suggest that the relationship between these strategies and psychopathology is relatively smaller (42, 51). However, in a recent meta-analysis, Schäfer et al. (52) found comparable effects of adaptive strategies to maladaptive ones in relation to depression and anxiety among adolescents.

In ER research, there has been a particular emphasis on rumination [defined as a stable tendency to focus passively and repetitively on distress-related feelings, their causes and consequences (55)], given that it is strongly associated with overall psychopathology (51). Maladaptive ruminative thoughts are usually distressing and can contain many overgeneralized, abstract (56), and self-critical thoughts (57) that are difficult to control. Therefore, it has been proposed that rumination may induce maladaptive behaviors (e.g., psychoactive substance use) that provide disengagement or escape from these thoughts (58). Based on this, it appears plausible to hypothesize that adolescents who are more prone to ruminate are at greater risk of developing problem gaming. In addition, for adolescent boys who are more likely to be engaged in problem gaming (59), the association between rumination and problem gaming may be much stronger than for adolescent girls who are less likely to be at risk for problem gaming.

Given this contextual empirical evidence, the present study i) examined whether ER strategies (particularly, rumination) were related to problem gaming and ii) tested whether gender moderates the relationship between rumination and problem gaming in adolescents. Recently, Aldao and colleagues (60) argued that the majority of the studies that targeted rumination only assessed this specific strategy instead of testing other ER strategies simultaneously. This narrow scope leaves open the question of how other ER strategies may relate to any psychological problems. Therefore, the present study assessed not just rumination but other ER strategies as well, especially putatively adaptive ones (such as positive reappraisal and acceptance) to test their potential protective effect on problem gaming. Since some studies suggest that specific game genres are more likely to relate to online problem gaming (6, 61, 62), the present study included game genre preference into the analyses to control for the effect of this variable.

## Methods

### Participants and Procedure

Survey data were collected as part of a nationwide cross-sectional study including 7th- to 14th-grade Hungarian students (altogether 27,728 classes and 654,921 students). In order to ensure the representativeness of the sample, a homogeneous stratified random sampling method was applied, based on regional characteristics (capital city and countryside), class type (primary general, secondary general, secondary vocational, and vocational classes), and grade (7th–14th), where the sampling unit was the class. The participants were asked to complete the surveys in the classroom within one class session. The sample characteristics therefore reflect the composition of participating classes. The data collection was supervised by trained research assistants, and no teaching staff were present. Participation in the study was voluntary and anonymous. No incentives were offered. All parents and students were issued with written informed consent letters.

The data were successfully collected in 200 classes (out of the selected 253), among 3,509 students (1,657 boys and 1,852 girls). The rate of missing data was 20%, which derived from the refusal of parents and/or participants, and pupil absences during the data collection period. In our representative study (*N* = 3,509), 14.6% reported no gaming activity in their lifetime, while another 4.7% of the sample did not provide an answer to online gaming use question. A total of 29.4% participants indicated lifetime use, but did not play in the past 12 months, while 51.3% reported online gaming in the previous 12 months (*N* = 1,799). Some participants (*N* = 153) who reported online gaming in the previous 12 months did not answer Problematic Online Gaming Questionnaire Short-Form (POGQ-SF) items; therefore, we excluded them from further analyses. Consequently, the final sample that was used in the further analyses comprised 1,646 students (mean age = 15.40 years, SD = 2.19 years; 62.9% boys).

The study was ethically approved by the Institution Review Board of ELTE Eötvös Loránd University, and the research was carried out in accordance with the Declaration of Helsinki.

### Measures

The short form of the Cognitive Emotion Regulation Questionnaire (CERQ-short) (63) was used to assess the set of cognitive ER strategies that individuals apply in response to stressful life events. The CERQ-short is a self-report 18-item instrument that quantifies ER *via* nine dimensions (each consisting of two items): i) self-blame, ii) other-blame, iii) rumination, iv) catastrophizing, v) putting into perspective, vi) positive refocusing, vii) positive reappraisal, viii) acceptance, and ix) planning. Theoretically, the nine strategies can be grouped into adaptive (putting into perspective, positive refocusing, positive reappraisal, acceptance and planning) or maladaptive (self-blame, other-blame, rumination, catastrophizing) ER strategies. Participants were asked to score the items (e.g., “I think I can learn something from the situation”) on a five-point Likert scale (1 = Almost never to 5 = Almost always). The sum of the scores belonging to each subscale was calculated and used in the analyses where higher scores indicated more usage of the specific strategy. The Hungarian adaptation of the long-version CERQ (64) showed good internal consistency (Cronbach’s α ranged from 0.68 to 0.88) and strong test–retest reliability (Pearson’s *r* = 0.58–0.88). In the present sample, the nine subscales had acceptable or good reliability (see **Table 2**).

Self-reported problem gaming was assessed using the POGQ-SF (59, 65). The original 18-item instrument (66) was shortened to 12 items (e.g., “When you are not gaming, how often do you think about playing a game or think about how would it feel to play at that moment?”; “How often do you feel depressed or irritable when not gaming only for these feelings to disappear when you start playing?”), maintaining the original six-factor structure (preoccupation, overuse, immersion, social isolation, interpersonal conflicts, and withdrawal). Participants were asked to indicate on a five-point Likert scale how often the items apply to themselves (1 = Never to 5 = Always), where higher scores reflected higher risk for online problem gaming. No reverse coded items were applied. The sum of the scores was calculated and used in the analyses. The POGQ-SF showed configural, metric, and scalar level gender invariance (59). In the study sample, the total score had excellent reliability (see **Table 2**).

In addition to the CERQ and the POGQ-SF, basic sociodemographic data (e.g., age and gender), gaming in the past 12 months, and game genre preference were collected. Game genre preference was collected by asking participants what type of online game they played primarily [i.e., strategy games, role-playing games, casual games, shooter games, MOBA (multiplayer online battle arena) games, other game types].

### Statistical Analysis

SPSS 25.0 (IBM) and MPlus 7.4 (67) statistical software were used for the analyses. First, the factor structure of the CERQ-Short Form was tested on the whole sample, since the psychometric properties of the short version had not previously been tested on a Hungarian adolescent sample. The original nine-factor structure was tested and the second-order factor structure (with adaptive and maladaptive strategies) with confirmatory factor analysis (CFA) was utilized. In CFA, maximum likelihood estimation robust to non-normality (MLR) was used. To evaluate the overall model fit, the present study used the conventional χ^2^ test and the more liberal indices such as comparative fit index (CFI), Tucker–Lewis Fit Index (TLI), and root mean square error approximation (RMSEA). CFI and TLI—which reflect the total variance accounted for by the model and indicate a fit relative to a null model—are expected to be above 0.9 to indicate acceptable fit of the model. Values below .05 for RMSEA, reflecting the variance of residuals, signify an excellent fit. Non-significant probability (*p* > .05) values of closeness of model fit using the RMSEA (Cfit of RMSEA) indicate acceptable model fit, although some statisticians argue for larger values, such as *p* > .5 (68). To compare the two alternative nested models, the Satorra–Bentler scaled χ^2^ difference test was also applied (69). Cronbach’s α values were calculated as indices of reliability of the subscales of the CERQ-Short and the POGQ-SF. To test the relationship between online gaming and ER strategies, linear regression analysis was used. Gender, age, and game genre preference were controlled for in this model. Additionally, the present study tested whether gender moderated the relationship between ER strategies, especially rumination and problem gaming. For exploratory purposes, we ran a series of analyses to test whether gender moderated the effect of other ER strategies on online problem gaming. Similarly, we also ran a series of exploratory analyses to test whether preferred game genre moderated the effect of ER strategies on online problem gaming (see **Supplementary Tables 1** and **2**). We used the adjusted *R* ^2^ and AIC (Akaike’s Information Criteria) values to decide about the inclusion of any other interaction terms in our regression analysis. Adjusted *R* ^2^ adjusts the *R* ^2^ for the number of independent variables (predictors) in the model. AIC as a model performance metric penalizes the inclusion of additional variables to a model. A smaller value of AIC indicates a better model fit.

## Results

### Factor Structure of the Cognitive Emotion Regulation Questionnaire–Short

The original nine first-order factor model and the second-order factor structure (with adaptive and maladaptive strategies) on the whole sample were tested. The nine first-order factor measurement model fitted the data well (χ^2^ = 766.215, df = 99, *p* < 0.001; RMSEA = 0.046 [0.043–0.049], cfit = 0.984, CFI = 0.954, TLI = 0.929), while the second-order factor model had a considerably worse fit to the data (χ^2^ = 1674.641, df = 125, *p* < 0.001; RMSEA = 0.062 [0.062–0.065], cfit < 0.001, CFI = 0.893, TLI = 0.869). The original nine-factor structure model yielded a superior fit compared to the second-order factor model (Satorra–Bentler Δχ^2^ = 871.945, df = 26, *p* < 0.0001). In the nine-factor model, all the factor loadings were above 0.66. Based on the CFA results, the nine ER strategies were used in the subsequent analyses; we did not group them into adaptive and maladaptive clusters.

### Descriptive Data

Participants reported the most involvement in shooter games (23.3%), with approximately equal proportions of the participants preferring strategy (14.3%), casual (14.7%), and online role-playing games (14.0%). The remaining gamers indicated a preference for MOBA games (10.4%) and other game genres (14.7%), with a further 8.5% not providing an answer. Significant gender differences were found in game genre preference (⶘χ^2^[6] = 364.401, *p* < 0.001). Among those who primarily played shooter or MOBA games, there were more boys, while casual games were preferred by girls (**Table 1**).

**Table 1:** Online problem gaming score as a function of game genre preference.

Preference for								
	Strategy games	Role-playing games	Shooter games	MOBA games	Casual games	Other game genres	Did not give an answer	Total sample
N	236	231	383	172	242	242	140	1646
Mean (SD)	19.17 (7.47)_a,b_	23.22 (8.51)_c,d_	22.83 (9.19)_c,d_	24.57 (9.28)_d_	17.26 (5.73)_a_	21.05 (8.92)_b,c_	22.67 (9.61)_c,d_	21.45 (8.74)
Gender: Male	61.0%_a_	68.4%_a_	86.4%_b_	84.3%_b_	16.1%_c_	53.3%_a_	63.6%_a_	62.9%

Different letters (a, b, c, d) in the same row represent significant (p < .05) difference between mean scores, whereas the same letters in the same row represent non-significant difference between mean scores according to the paired post hoc Tukey test of one-way ANOVA or the paired chi-square tests. MOBA, multiplayer online battle arena.

A one-way ANOVA was performed to examine the effect of game genre preference on the online problem gaming score. Results showed that the game genre preference had a significant effect on the POGQ-SF score (*F*_6,1639_ = 20.735, *p* < 0.001). Those who preferred casual games or strategy games had significantly lower online problem gaming scores compared to those who preferred shooter, role-playing, or MOBA games (see **Table 1**). Given these differences, a binary variable was created for game genre preference, which included those who preferred shooter, role-playing, or MOBA games and those who preferred strategy, casual, or other game genres. This binary variable was used in the subsequent analyses.

Means and standard deviations of the psychological scales are presented in **Table 2**. Males and females differed from each other on all scales except positive refocusing and acceptance. Males had higher score on online problem gaming and reported more frequent use of other blaming in times of stress, while females reported more use of self-blame, rumination, catastrophizing, putting into perspective, planning, and positive reappraisal. The effect size for gender differences in online problem gaming was moderate in magnitude (Cohen’s *d* above 0.5); in self-blame and in rumination, it was small (Cohen’s *d* above 0.2); and for other ER strategies, the gender differences were negligible (Cohen’s *d* below 0.2). Age did not correlate with online problem gaming scores (*r* = 0.003, *p* = 0.92) or with ER strategies (*r* values were between −0.04 and 0.05; *p* > 0.05). ER strategies were correlated with each other positively, and the correlation coefficients ranged between 0.05 and 0.54 (**Table 3**), with most of them being moderate (between 0.30 and 0.50).

**Table 2:** Means, standard deviations, and effect sizes (Cohen’s *d*) by gender with Cronbach’s α.

Scales	Mean (SD)	Cronbach α	Males	Females	*t*/*d*	Cohen’s *d*
POGQ-SF total score	21.45 (8.74)	0.90	23.22 (9.07)	18.44 (7.22)	11.774***	0.58
CERQ Self-blame	5.33 (2.02)	0.67	5.15 (1.98)	5.62 (2.05)	4.328***	0.23
CERQ Acceptance	6.28 (2.05)	0.71	6.22 (2.11)	6.37 (1.95)	1.341	0.07
CERQ Rumination	5.99 (2.14)	0.78	5.67 (2.12)	6.50 (2.09)	7.340***	0.39
CERQ Positive refocusing	4.94 (2.12)	0.72	4.90 (2.13)	5.01 (2.10)	0.942	0.05
CERQ Planning	6.06 (2.05)	0.67	5.93 (2.08)	6.27 (1.99)	3.029**	0.17
CERQ Positive reappraisal	6.21 (2.06)	0.61	6.12 (2.08)	6.35 (2.01)	2.153*	0.11
CERQ Putting into perspective	5.59 (2.02)	0.64	5.50 (2.03)	5.74 (2.00)	2.239*	0.12
CERQ Catastrophizing	4.57 (2.12)	0.77	4.42 (2.07)	4.81 (2.18)	3.396***	0.18
CERQ Other blame	4.00 (1.82)	0.67	4.15 (1.86)	3.78 (1.73)	3.748***	0.20

POGQ-SF, Problematic Online Gaming Questionnaire Short Form; CERQ, Cognitive Emotion Regulation Questionnaire; SD, standard deviation; *p < .05; **p < .01; ***p < .001.

**Table 3:** Correlations between problem gaming and cognitive emotion regulation strategies.

	2.	3.	4.	5.	6.	7.	8.	9.	10.
1. POGQ-SF total score	.20	*.01*	.20	.14	*.08*	*.03*	.10	.28	.22
2. CERQ Self-blame	1	.33	.54	.20	.41	.30	.29	.50	.18
3. CERQ Acceptance		1	.42	.25	.39	.53	.34	.16	*.05*
4. CERQ Rumination			1	.27	.45	.46	.34	.48	.16
5. CERQ Positive Refocusing				1	.31	.35	.41	.28	.32
6. CERQ Planning					1	.51	.50	.28	.19
7. CERQ Positive Reappraisal						1	.45	.19	.10
8. CERQ Putting into perspective							1	.21	.24
9. CERQ Catastrophizing								1	.40
10. CERQ Other blame									1

POGQ-SF, Problematic Online Gaming Questionnaire Short Form; CERQ, Cognitive Emotion Regulation Questionnaire. All the correlational coefficients are significant except those in italics after Bonferroni correction (p < .00061).

### Relationship Between Emotion Regulation Strategies and Online Problem Gaming

First, linear regression analysis was applied to examine whether ER strategies explained the variance in online problem gaming total score. Gender, age, and game genre were controlled for in the analysis. Game genre was based on both theoretical consideration and the empirical data (see above). More specifically, a variable with two values was created (0 indicated preference for either shooter, role-playing, or MOBA games; 1 indicated preference for any other games). As expected, the results showed that maladaptive strategies, namely, self-blame, rumination, catastrophizing, and other blame, were positively related to online problem gaming. For the adaptive strategies, higher scores on positive reappraisal were associated with lower online problem gaming score, while positive refocusing and planning were positively related to online problem gaming (see **Table 4**). The final model (*F*_12,1267_ = 27.26, *p* < 0.001) explained 20.5% of the total variance of the POGQ-SF total score.

**Table 4:** Standardized regression weights between online problem gaming and emotion regulation strategies after controlling for age, gender, and game preference.

	Standardized β	Significance	*R* ^2^/Adjusted *R* ^2^	AIC
*Main effects*				
Gender	−.214	<.001	.205/.198	5,247.259
Age	−.051	.045		
Game genre preference	−.189	<.001		
CERQ Self-blame	.081	.013		
CERQ Acceptance	−.018	.567		
CERQ Rumination	.078	.026		
CERQ Positive Refocusing	.077	.009		
CERQ Planning	.079	.016		
CERQ Positive Reappraisal	−.086	.011		
CERQ Putting into perspective	−.016	.599		
CERQ Catastrophizing	.079	.018		
CERQ Other blame	.142	<.001		
*Main effects + Interaction between gender and rumination*				
Gender	−.005	.947	.210/.202	5,241.314
Age	−.051	.042		
Game genre preference	−.187	<.001		
CERQ Self-blame	.083	.010		
CERQ Acceptance	−.018	.558		
CERQ Rumination	.138	.001		
CERQ Positive Refocusing	.076	.011		
CERQ Planning	.076	.020		
CERQ Positive Reappraisal	−.088	.009		
CERQ Putting into perspective	−.021	.508		
CERQ Catastrophizing	.082	.013		
CERQ Other blame	.134	<.001		
Gender × CERQ rumination	−.240	.005		
*Main effects + Interaction between gender and rumination + Interaction between game genre and other blame*				
Gender	−.022	.777	.218/.209	5,230.381
Age	−.047	.059		
Game genre preference	.017	.787		
CERQ Self-blame	.084	.009		
CERQ Acceptance	−.020	.509		
CERQ Rumination	.128	.002		
CERQ Positive Refocusing	.071	.016		
CERQ Planning	.070	.031		
CERQ Positive Reappraisal	−.085	.011		
CERQ Putting into perspective	−.020	.523		
CERQ Catastrophizing	.081	.014		
CERQ Other blame	.217	<.001		
Gender × CERQ rumination	−.222	.009		
Game genre × CERQ other blame	−.233	<.001		

CERQ, Cognitive Emotion Regulation Questionnaire; Gender: 0, boys; 1, girls; game genre preference: 0 indicated preference for either shooter, role-player, or MOBA games, and 1 indicated preference for any other games; AIC, Akaike’s Information Criteria.

### Relationship Between Rumination and Online Problem Gaming Is Moderated by Gender

**Figure 1:**
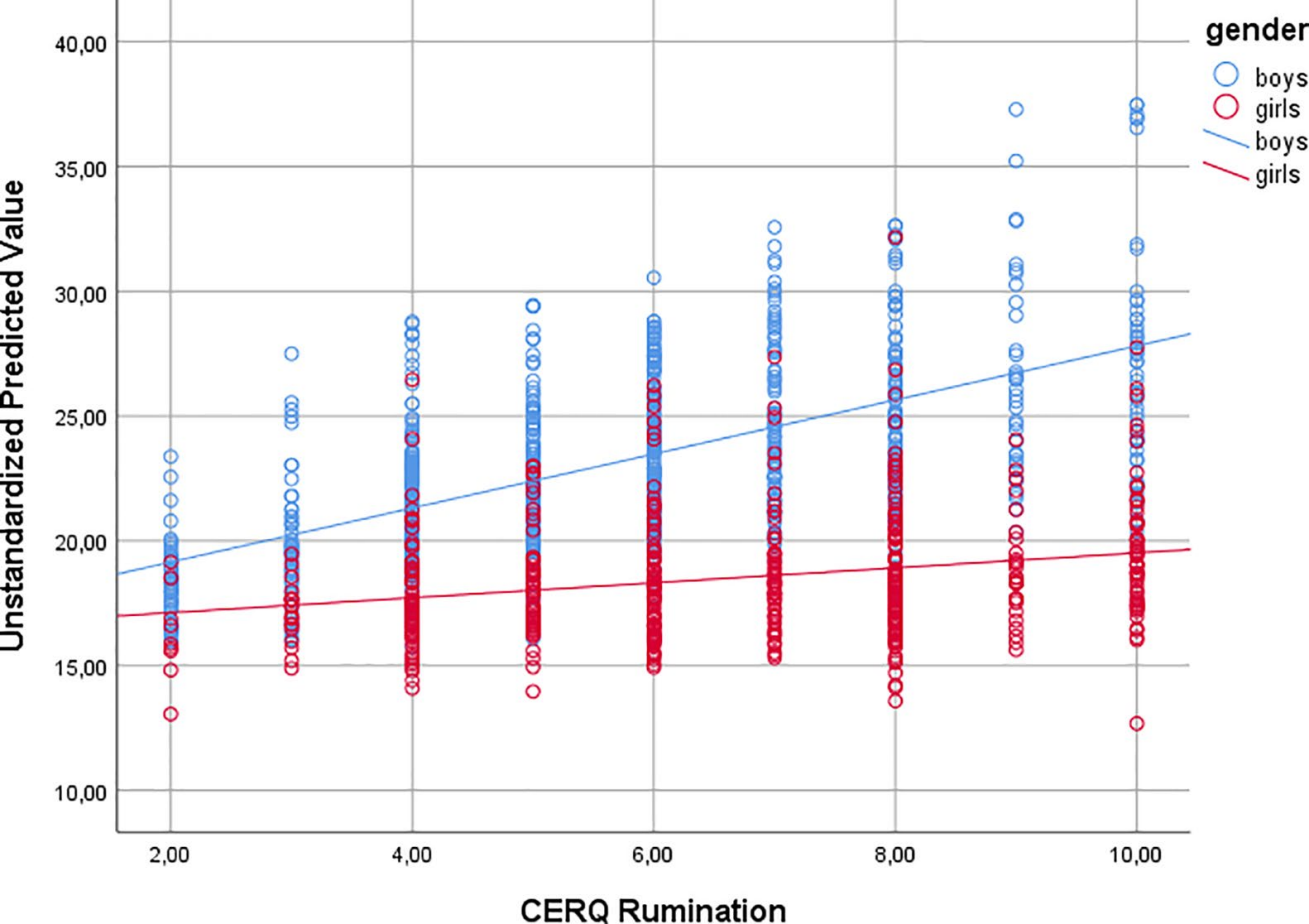
The relationship between rumination and online problem gaming by gender.

Finally, to understand gender differences in explaining online problem gaming, a moderation analysis was performed. The analysis added the interaction term (gender × rumination) to the previous linear regression analysis, and it yielded to be a significant independent variable (standardized β = −0.240, *p* = 0.005) and was associated with significant R2 change (F change = 7.882, *p* = 0.005). However, the explained variance did not change substantially (0.5%). The moderation effect is shown in **Figure 1**. It can be seen that as rumination scores increased, the online problem gaming score also increased but primarily among males. A simple correlational analysis between rumination and online problem gaming by gender also supported this result (*r*_boys_ = 0.25, *p* < 0.001; *r*_girls_ = 0.08, *p* = 0.049).

For exploratory purposes, we ran a series of analyses to test whether gender moderated the effect of other ER strategies on online problem gaming, first including only one interaction term in the regression model each time. We found that gender × catastrophizing interaction contributed to the variance of online problem gaming significantly (standardized β = −0.150, *p* = 0.023). Similarly, we also ran a series of analyses to test whether preferred game genre moderated the effect of ER strategies on online problem gaming, first including only one interaction term in the regression model each time. Four interaction terms proved to be significant: the catastrophizing × game genre (standardized β = −0.214, *p* = 0.001), other blame × game genre (standardized β = −0.243, *p* < 0.001), rumination × game genre (standardized β = −0.178, *p* = 0.028), and self-blame × game genre (standardized β = −0.150, *p* = 0.048) interaction terms (see **Supplementary Table 1**).

**Figure 2:**
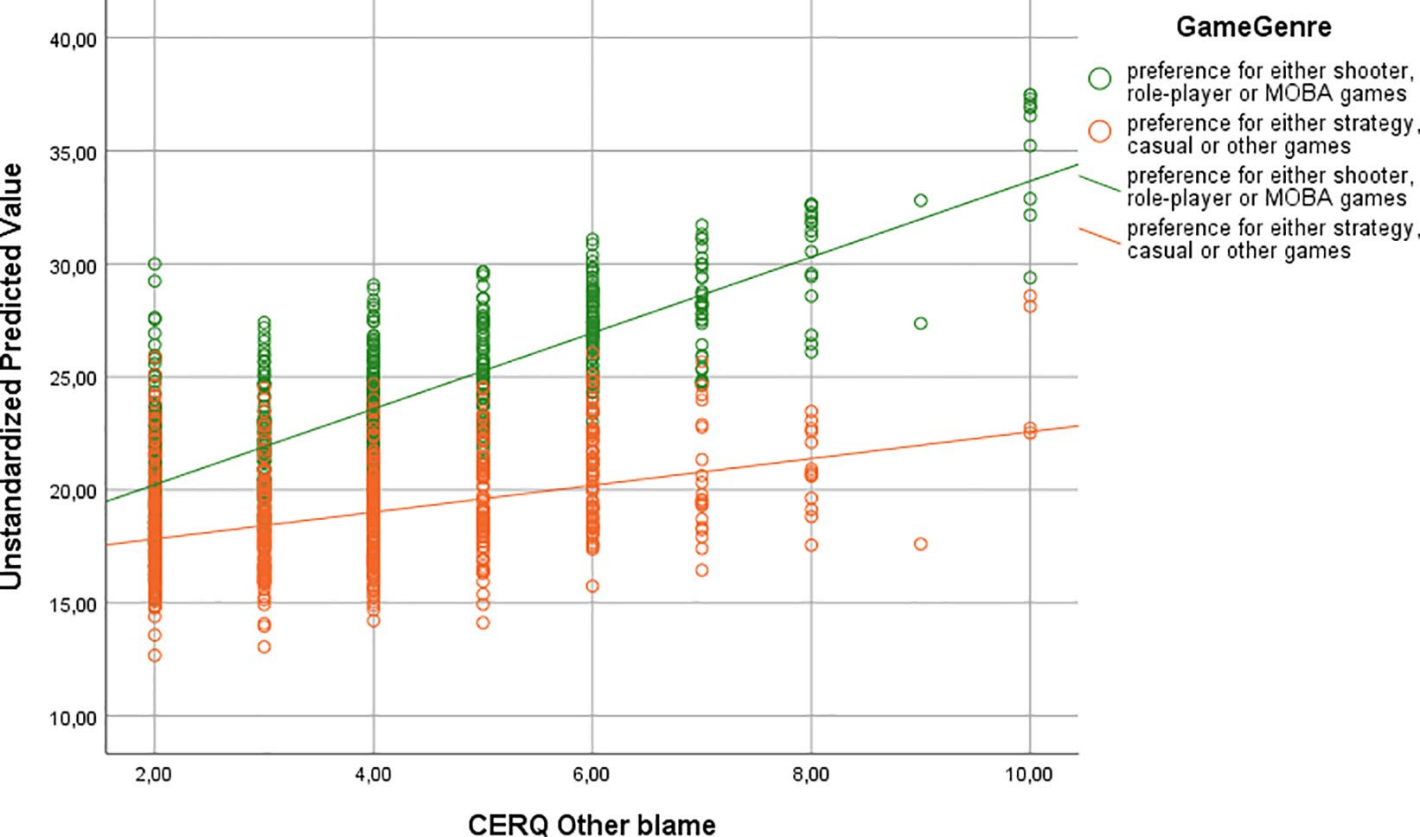
The relationship between other blame and online problem gaming by game genre.

In the next step, we added these interaction terms to our model to test whether adjusted *R* ^2^ and AIC would change. Based on these additional analyses (see **Supplementary Table 2**), our third model also includes the interaction between game genre and other blame ER strategy (**Table 4**). The inclusion of this interaction was associated with significant *R* ^2^ change (*F* change = 12.846, *p* < 0.001). The effects of other blame were stronger among those who preferred shooter, role-playing, or MOBA games than among those who preferred strategy, casual, or other game genres. A simple correlational analysis between self-blame and online problem gaming by game genre also supported this result (*r* = 0.32, *p* < 0.001 and *r* = 0.14 *p* < 0.001, respectively). The moderation effect is shown in **Figure 2**.

## Discussion

Hemenover and Bowman (70) recently proposed extending the scope of research on gaming to include the emotion and ER field. Now—and based on the results of the present study—the present authors also argue that ER is a useful framework to study online problem gaming. It was particularly expected that rumination would be associated with online problem gaming, but our aim was to test the potential effect of other putatively maladaptive and adaptive strategies as well. In line with the present conceptualization, online problem gaming was treated as a continuum and found—as logically expected—that it was positively associated not just with rumination but with the other maladaptive cognitive ER strategies. The results also support the contention that the use of positive reappraisal has a beneficial effect on functioning given that it was inversely related to online problem gaming when other strategies were controlled for in the analysis. In addition, the present study highlighted that the relative predictive value of rumination for online problem gaming varied for boys and girls.

Studies elsewhere have demonstrated that the use of cognitive reappraisal assessed by self-reports (71) or induced by experimental manipulations (72) is associated with higher levels of psychosocial well-being. Additionally, prospective studies carried out with adolescents have shown that cognitive reappraisal can act as a buffer against the negative impacts of adverse life events (73, 74). The results of the present study extend these previous findings and suggest that trait reappraisal could also be a protective factor against online problem gaming among adolescents. A previous study using a small sample comprising young adults with IGD showed that the IGD group reported less use of positive reappraisal compared to controls (75).

During reappraisal, individuals transform the meaning of situations by changing the way they think about them (76). Although there are different tactics concerning how to do it (77, 78), reinterpreting the emotional stimulus or the event in more positive terms (e.g., “I think I can learn something from the situation”)—as the CERQ subscale defines this cognitive change strategy (79)—appears to be effective in modulating experiential (self-reported emotional experience) and behavioral (observer-reported emotional expression) aspects of negative emotions (78), therefore contributing to better psychological health. Moreover, neuroimaging studies have found that individuals with higher tendency to use reappraisal utilize frontal regions to a higher extent and amygdala to a lesser extent when they are instructed to reappraise negative stimuli (80) or when just simply processing them (81). Results on the downregulation of amygdala activity highlight that a decrease in negative emotions after positive reappraisal can be detected not just at a subjective level but also at a neural level.

It is also worth noting that findings of experimental studies suggest that the timing of reappraisal (82) or emotional intensity (83) may determine whether such a strategy is an adaptive and/or effective one in modulating emotions. For example, it is much easier to reinterpret a low arousing stimulus/event than a high arousing one (84), and reappraising a negative stimulus is more effective if this strategy initiated early in the emotion generating process (e.g., 82, 85). In addition, the characteristics of a specific situation (stressor) may also shape the costs or benefits of this strategy (86, 87). For example, in case of uncontrollable stressors, reappraising can weaken the negative impact of the stressor (73, 88). Conversely, when the stress is controllable, higher use of reappraisal may contribute to poorer psychological health (88). Further studies need to understand how positive reappraisal and its variants/tactics such as reinterpretation or psychological distancing (89) are associated with online problem gaming prospectively, especially during childhood and adolescence when maturation of brain areas involved in ER is still ongoing (90).

Interestingly, the putatively adaptive ER strategies (i.e., positive refocusing and planning) were positively related to online problem gaming. It appears logic that planning itself—defined as thinking about what to do and how to solve the problem (91)—without any action does not guarantee the success of ER. Indeed, studies using the CERQ among adolescents did not find any relationship between planning and internalizing or externalizing symptoms (92) or between planning and anxiety symptoms when depression was controlled for (93).

The adaptive or maladaptive nature of positive refocusing—thinking about positive things instead of thinking about the actual negative event—is still questionable. Positive refocusing is an event-avoiding (92) active strategy (78). For instance, in adolescents, it has been found to be a unique predictor of externalizing symptoms (92). Active distraction from negative events or distress *via* positive refocusing can downregulate negative emotions in the short term (78) and has been negatively associated with depressive mood among children and adolescents in cross-sectional studies (93–95). However, if refocusing serves as an avoidance of private events such as emotional experiences, thoughts, and memories (termed experiential avoidance) (96), then it may just exacerbate or prolong negative emotions in the long term (97), thus contributing to elevated levels of psychopathological symptoms (35, 36). These results strongly suggest that ER goals are likely to determine whether any distraction-like strategies contribute to better or poorer functioning. Regarding online gaming, it is tempting to hypothesize that gaming motives may moderate the relationship between positive refocusing and online problem gaming. However, since gaming motives were not assessed in the present study, future research should include such variables.

Acceptance—treated frequently as the opposite of experiential avoidance (98) or as a component of mindfulness (99, 100)—has been proved to be positively associated with psychological health in both adult (101) and adolescent samples (102, 103). However, in the present study, there was no relationship between this strategy and online problem gaming. This might be because the CERQ-Short uses items relating to accepting situations rather than accepting emotions. For instance, in a recent study by Ford and colleagues (104), habitual use of acceptance was related to better psychological health if it referred to accepting mental experiences. Accepting the stressful situations showed no relationship with psychological well-being, life satisfaction, or depressive symptoms in Ford et al.’s study (104).

All the putatively maladaptive ER strategies—rumination, self-blame, other blame, and catastrophizing—had a positive association with online problem gaming in the present study. This supported previous findings on the relationship between maladaptive strategies and poorer psychological functioning in adolescents (52). It was particularly expected that rumination would relate to online problem gaming because its habitual use has been strongly associated with overall psychopathology (51). The main effect of rumination is supported by the data in the present study, but our findings also pointed out that other strategies could be relevant in the context of online problem gaming. Our results specifically suggest that handling stress-related negative feelings and emotions with using any putatively maladaptive cognitive ER strategies is a risk factor for online problem gaming.

It was also found that gender moderated the relationship between rumination and online problem gaming. Adolescent boys had higher score on the scale measuring online problem gaming compared to girls confirming prior research (4, 59). In addition, rumination was related to online problem gaming among boys, but the association was weak among girls. It has been well documented—and the results here are in line with this—that rumination is higher among women and that this gender difference is already present in adolescence (105). The predominance of females with depression and anxiety disorders has therefore been (partially) explained by more ruminative tendencies among females compared to males (55, 106). Based on the results of the present study, it is tempting to suggest that high ruminative tendency in men creates a vulnerability factor for pathologies that are associated with behaviors offering an escape from (thinking about) real-life problems. However, it is important to note that more boys reported online gaming in the study than girls, and this difference in the number of users may have influenced the findings regarding rumination. Thus, it would be interesting to replicate the study among female gamers to see whether ruminative tendencies are related to online problem gaming among them.

Further studies are also needed to address whether escapism moderates the relationship between rumination and online problem gaming and whether such moderation is invariant across gender. Of interest, the present authors treated rumination as a maladaptive ER strategy, but rumination is a multidimensional construct. It is likely that brooding, defined as a tendency to passively dwell on negative emotions (107), would be more strongly related to problem gaming than reflective pondering, defined as a more purposeful self-reflective response to understanding and solving problems (107).

The present study found that those adolescents who reported a preference for shooter, role-playing, and MOBA games had higher scores on the POGQ-SF, while those who preferred casual or strategy games had lower POGQ-SF scores. This finding is in line with previous studies and supports the finding that some gaming genres appear to be more problem-inducing than others. Massively multiplayer online role-playing games (MMORPGs) offer the possibilities for role-playing, progression, action, and social interaction in a persistent virtual world (108), and may relate to motivations such as escapism (which has associations with problem gaming). Online shooter games (109) and MOBA games (110) have also been associated with gaming-related problems, and this may be due to structural and situational factors such as being fast-paced, action-oriented, and socially interactive (111). However, it should also be noted that motivations underlying gaming (62, 112, 113) and personality factors (112) may influence these relationships with problem gaming. In addition, our exploratory analyses yielded an interesting result. We found that the effect of other blame on online problem gaming was moderated by the game genre preference. The effects of other blame were stronger among those who preferred shooter, role-playing, or MOBA games than among those who preferred strategy, casual, or other game genres. In certain game genres, especially in MOBA games, shooters, and role-playing games [in PvE (person vs. environment) type quests], team performance is highly dependent on the personal performance of team members. Therefore, blaming others within one’s own team in the case of lost matches/quests/raids is characteristic of these game genres, especially MOBA games (114, 115). This might explain why other blame ER strategy has a stronger effect among players who preferred these game genres. There were students, however, who did not report their preferred game genre; thus, they were not included in these interaction analyses and this might distort the results.

The results presented here support the theoretical structure of CERQ-Short in an adolescent representative sample and is the same as the original length CERQ, thus confirming the original nine-factor structure of the instrument. However, the nine strategies were not grouped into the proposed higher-order (i.e., maladaptive and adaptive) factors. Taking into account that context strongly determines whether a specific strategy is an adaptive one or not (86), these results appear to be unsurprising. In addition, individuals may use different strategies in different contexts (e.g., rumination after peer rejection but positive reappraisal after minor failure). Consequently, a positive relationship between the use of adaptive and maladaptive strategies would be expected. Indeed, there were significant moderate and positive relationships between the adaptive and maladaptive ER strategies, which also prevented the identification of the proposed two-factor higher-order solution.

### Limitations and Future Research

The use of a cross-sectional design did not allow any inference that ER plays a pivotal role in the development of problem gaming. However, it is worth mentioning that in a 2-year prospective study in the United States, the ability to deal with stress predicted who developed pathological gaming among adolescents (24). More longitudinal studies are needed to evaluate whether regulation of negative (or even positive) emotions predicts the development of online problem gaming. Further studies should also assess other gaming-related constructs associated with problem gaming such as beliefs or metacognitions about gaming (116), gaming use expectations (117), as well as motives for gaming (21) to estimate the contribution of ER more precisely. Similarly, including other individual risk factors for problem gaming such as impulsivity (118), sensation seeking (119), peer influence (117), and/or parental factors (120) would also be beneficial.

However, time spent gaming itself is not considered to be a reliable predictor of problem gaming (25, 27), although inclusion of this variable may have improved the analysis. Similarly, other variables were not assessed including depressive symptoms, anxiety, and/or other psychopathological problems, and such variables are reliably related to both ruminative tendencies (107, 121) and online problem gaming (21). It is plausible to hypothesize that the relationship between psychopathological symptoms and online problem gaming is mediated or even moderated by ruminative tendencies or by other strategies. For instance, catastrophizing has been demonstrated to relate to anxiety symptoms (93). Based on this finding, it is also tempting to hypothesize that different putatively maladaptive strategies have different “pathways” into online problem gaming.

ER was assessed with self-reports (and are subject to well-known biases), but application of other methods such as a daily diary or an experience sampling method would be useful to demonstrate that everyday ER, negative affect, motives, and gaming are interrelated, providing more ecologically valid data. It would also be worth testing how the interaction between adaptive and maladaptive ER strategies affects psychological health. For instance, in a daily diary study, it was shown that reappraisal moderated the relationship between rumination and depressive and social anxiety symptoms (122). Consequently, it appears plausible to hypothesize that patterns of rumination and positive reappraisal may relate to online problem gaming differently. Latent profile analysis would be a good candidate to answer this question. Finally, it is worth mentioning that ER strategies were assessed generally and did not take into account how specific emotions—for example, anger, sadness, fear, or positive emotions such as joy or pride—are regulated (123).

## Conclusion

The results of the present study draw attention to ER, namely, habitual ER strategies used in times of stress in online problem gaming. It appears to be important given that ER can be targeted and improved during development, and can even be altered in adulthood *via* different interventions and psychotherapies. Our findings entail important implications for mental health prevention. Prevention programs that promote positive reappraisal and help to understand negative consequences of frequent use of maladaptive ER strategies such as rumination or catastrophizing could be useful in developing healthier ways of handling everyday negative emotions in adolescence. It is also worth noting that negative emotions are frequently elicited by recalling personally relevant negative events that happened in the past or in the near past. Cognitive and emotional responses to these thoughts will influence the intensity and duration of negative affect. Thus, prevention efforts that focus on adaptive ways of elaborating (processing) negative events can prevent unproductive rumination and the development of psychopathologies, including IGD as well.

At the same time, the relationship between online gaming and ER can be thought of the other way around. Recently, in their systematic review of videogames for ER, Villani et al. (124) came to the conclusion that (specific types of) videogames may be promising tools for improving the ER skills of gamers. Taking this a step further, Dore et al. (125) proposed a personalized investigation of ER *via* understanding how the individual, the situation, and strategies interact with each other and thus contribute to successful or unsuccessful regulation of emotion.

The understanding of gender-related behavioral and neural factors in online gaming is considered to be an important public health issue (126). The findings of the present study suggest that trait rumination among boys might be such a vulnerability factor for online problem gaming. Understanding gender-specific trajectories in online gaming, if any, requires longitudinal studies. These studies may then shed light on whether common or distinct factors predict the problematic use of different online activities (e.g., gaming, social networking, and watching porn) and whether these factors are gender specific.

## Author Contributions

ZD and BP conceived and designed the study. AM was responsible for data collection. Data analysis was performed by GK with special assistance from NK, AG, and OK. GK, NK, MG, OK, and ZD contributed to the interpretation of the data. GK, NK, and OK wrote the first draft of the manuscript, and all authors provided critical revision to its further development. MG oversaw the final manuscript editing. All authors read and approved the final manuscript.

## Funding

This study was supported by the Hungarian National Research, Development and Innovation Office (Grant No. K111938, KKP126835). This work was completed in the ELTE Institutional Excellence Program (783-3/2018/FEKUTSRAT) supported by the Hungarian Ministry of Human Capacities. Financial support was received from the Szerencsejáték Plc, which is the 100% state-owned national gambling provider in Hungary. The funding institutions had no role in the study design; the collection, analysis, and interpretation of the data; the writing of the manuscript; or the decision to submit the paper for publication. The preparation of this article for GK, NK, and AG was supported by the MTA-SE-NAP B Genetic Brain Imaging Migraine Research Group, Hungarian Academy of Sciences, Semmelweis University (Grant No. KTIA_NAP_13-2-2015-0001); by the Hungarian Brain Research Program (Grant No. 2017-1.2.1-NKP-2017-00002); and by the Hungarian National Research, Development and Innovation Office (Grant No. FK128614). NK was supported by the ÚNKP-18-3-III-ELTE-495 New National Excellence Program of the Ministry of Human Capacities.

## Conflict of Interest Statement

The authors declare that the research was conducted in the absence of any commercial or financial relationships that could be construed as a potential conflict of interest.
