# Supplementary material for: The Role of Cognitive Emotion Regulation Strategies in Problem Gaming Among Adolescents: A Nationally Representative Survey Study
Source: Front Psychiatry. 2019 Apr 29;10:273. doi: 10.3389/fpsyt.2019.00273 (PMC6501698; doi:10.3389/fpsyt.2019.00273)
Supplement: Supplementary file 1 [file DataSheet_1.docx]

Supplementary Material

For exploratory purposes, a series of linear regression analyses was performed to test whether gender moderated the effect of emotion regulation (ER) strategies (other than rumination) on online problem gaming. Similarly, we also ran a series of analyses to test whether preferred game genre moderated the effect of ER strategies on online problem gaming. In all models age, gender, preferred game genre and the main effects of all ER strategies were included. Only one interaction term was included in the regression model each time. Standardized betas and related significance levels are presented in Supplementary Table 1.

Table S1. Standardized regression weights of interaction terms (gender x emotion regulation strategies and preferred game genre x emotion regulation strategies) explaining online problem gaming score.

|  | interaction with gender | | interaction with preferred game genre | |
| --- | --- | --- | --- | --- |
|  | Standardized beta | Significance | Standardized beta | Significance |
| CERQ Self-blame | -.073 | .353 | -.150 | .048 |
| CERQ Acceptance | .013 | .884 | .007 | .935 |
| CERQ Rumination | -.240^*^ | .005 | -.178 | .028 |
| CERQ Positive Refocusing | -.100 | .152 | -.132 | .057 |
| CERQ Planning | -.045 | .600 | -.150 | .075 |
| CERQ Positive Reappraisal | -.018 | .833 | -.012 | .891 |
| CERQ Putting into perspective | -.056 | .484 | -.090 | .258 |
| CERQ Catastrophizing | -.150 | .023 | -.214 | .001 |
| CERQ Other blame | -.121 | .056 | -.243 | <.001 |

Note: dependent variable was online problem gaming score. CERQ: Cognitive Emotion Regulation Questionnaire; Gender: 0-boys, 1-girls; Game genre preference: 0 indicated preference for either shooter, role-player or MOBA games, and 1 indicated preference for any other games; *this interaction was included in our main analysis based on theoretical consideration and empirical findings presented in the main text of the manuscript

In the next step we added the significant interaction terms to our original model (M1) including age, gender, preferred game genre and the main effects of all ER strategies, and the interaction term of gender X rumination. We tested how the new independent variable would modify adjusted R-squared and AIC (Akaike’s Information Criteria) values (M2-M6). Both indices can guide the model choice when additional variables are included in the model. In Supplementary Table 2 these indices are shown.

Table S2. Regression model metrics.

|  | adjusted R-squared | AIC |
| --- | --- | --- |
| M0 | .198 | 5247.259 |
| M1 | .202 | 5241.314 |
| M2 (gender x catastrophizing) | .202 | 5242.124 |
| M3 (game genre x rumination) | .202 | 5242.081 |
| M4 (game genre x catastrophizing) | .206 | 5235.339 |
| M5 (game genre x other blame) | .209 | 5230.381 |
| M6 (game genre x self-blame) | .203 | 5241.237 |

Note: M0: regression model including age, gender, preferred game genre and the main effects of all ER strategies; M1: all variables included in M0 plus gender x rumination interaction term; IAC: Akaike’s Information Criteria.

Based on these additional analyses two interaction terms (game genre x catastrophizing and game genre x other blame) proved to be significant (standardized beta: -0.186 p=0.005 and -0.233 p=0.000, respectively) and the AIC values for these models also decreased, however adjusted R-squared values just slightly increased. Thus, we added these interaction terms to our M1 model. In this case, the game genre x self-blame interaction proved to be significant (standardized beta: -0.186 p=0.008), but the game genre x catastrophizing was a non-significant predictor (standardized beta: -0.106 p=0.142). Based on the result, we decided to add only the game genre x self-blame interaction to our M1 model (see the main text for further information).
